# Conserved Function of Core Clock Proteins in the Gymnosperm Norway Spruce (*Picea abies* L. Karst)

**DOI:** 10.1371/journal.pone.0060110

**Published:** 2013-03-28

**Authors:** Anna Karlgren, Niclas Gyllenstrand, Thomas Källman, Ulf Lagercrantz

**Affiliations:** 1 Dept. of Plant Ecology and Evolution, Evolutionary Biology Center, Uppsala University, Uppsala, Sweden; 2 Dept. of Plant Biology and Forest Genetics, Uppsala Biocenter, Swedish University of Agricultural Sciences, Uppsala, Sweden; University of Massachusetts Amherst, United States of America

## Abstract

From studies of the circadian clock in the plant model species Arabidopsis (*Arabidopsis thaliana*), a number of important properties and components have emerged. These include the genes *CIRCADIAN CLOCK ASSOCIATED 1* (*CCA1*), *GIGANTEA* (*GI*), *ZEITLUPE* (*ZTL*) and *TIMING OF CAB EXPRESSION 1* (*TOC1* also known as *PSEUDO-RESPONSE REGULATOR 1 (PRR1)*) that via gene expression feedback loops participate in the circadian clock. Here, we present results from ectopic expression of four Norway spruce (*Picea abies*) putative homologs (*PaCCA1, PaGI, PaZTL* and *PaPRR1*) in Arabidopsis, their flowering time, circadian period length, red light response phenotypes and their effect on endogenous clock genes were assessed. For *PaCCA1-ox* and *PaZTL-ox* the results were consistent with Arabidopsis lines overexpressing the corresponding Arabidopsis genes. For *PaGI* consistent results were obtained when expressed in the *gi2* mutant, while *PaGI* and *PaPRR1* expressed in wild type did not display the expected phenotypes. These results suggest that protein function of *PaCCA1, PaGI* and *PaZTL* are at least partly conserved compared to Arabidopsis homologs, however further studies are needed to reveal the protein function of *PaPRR1*. Our data suggest that components of the three-loop network typical of the circadian clock in angiosperms were present before the split of gymnosperms and angiosperms.

## Introduction

The circadian clock is the internal time keeping mechanism that helps a wide range of organisms including plants, animals, fungi and bacteria to control different physiological and metabolic processes in synchronization with the daily rhythms in the environment. This clock is defined as a system that sustains an endogenous rhythm of ∼24 hours, which persists in constant conditions. It can be reset and entrained from external environmental cues such as light and maintains a relatively constant rhythm over a broad range of temperatures [1].

It has been proven beneficial for plants to match their circadian rhythm with the onset of day and night [2], [3], allowing clock controlled processes such as leaf movement, hypocotyl expansion, and gene expression ([4] and references therein) to be scheduled at the most appropriate time point. The circadian clock is also a key component in photoperiodic responses, including induction of flowering and control of annual growth rhythm [5].

In the gymnosperm Norway spruce (*Picea abies* L. Karst) and other conifers, photoperiod is an important regulator of the annual growth cycle [6]. It is crucial for the tree to end the growth period and enter dormancy in time to avoid frost damage. Growth cessation and bud set is dependent on a critical night length, which displays a steep cline ranging from about two hours in the north to 6–7 hours in the more southern populations [6]. Unlike temperature, photoperiod and night length remain the same over years enabling the trees to adapt to their local environment. This response requires an internal time keeping mechanism and interestingly recent studies show that putative candidate genes from the photoperiodic pathway, including candidates from the circadian clock, are involved in the control of clinal variation for bud set in Norway spruce [7], [8]. It is thus important to unravel the mechanisms controlling circadian rhythms and photoperiodic responses in conifers, as well as the functional role of individual genes in the pathway.

Functional studies of the plant circadian clock have until recently been focused on the model species Arabidopsis (*Arabidopsis thaliana*). The circadian system has been shown to regulate a multitude of processes, ranging from germination to flowering. Gene expression studies have highlighted the importance of circadian control since about 30% [9] of the Arabidopsis transcriptome seems to be under the influence of the circadian clock. Most of these genes are so called output genes, which are controlled by the clock, but do not participate in the actual clock function. Based on current understanding there is a limited set of genes involved in creating and maintaining a circadian rhythm and a few gene families have emerged as key components in Arabidopsis.

One family contains the Myb-domain transcription factors *CIRCADIAN CLOCK ASSOCIATED 1* (*CCA1*) [10] and *LATE ELONGATED HYPOCOTYL* (*LHY*) [11]. Another family, named *PSEUDO-RESPONSE REGULATOR* (*PRR*), contains five genes, *TIMING OF CAB EXPRESSION1 (TOC1)*/*PRR1* and *PRR3, 5*, *7* and *9* [12], [13]. These two gene families together with other components are currently believed to participate in three interlocked feedback loops [14], [15]. The first identified loop consists of *CCA1*, *LHY* and *TOC1*, where CCA1 and LHY bind directly to an evening element at the *TOC1-*promoter and inhibit its expression [16]. Until recently, TOC1 was in turn believed to induce the expression of *CCA1* and *LHY*, possibly via an unknown component X, closing the first loop [16], [17]. However, new findings show that TOC1 represses *CCA1* and *LHY* expression [18]–[20], which alters the earlier proposed model. Huang *et al.* [19] also showed that TOC1 inhibit transcription of other important clock genes such as *PRR7, PRR9, GI* (*GIGANTEA*) [21], [22], *TOC1, ELF4* (*EARLY FLOWERING 4*) [23] and *LUX* (*LUX ARRHYTHMO* also know as *PHYTOCLOCK 1*) [24], [25], connecting the morning and evening loops described below. *CCA1* and *LHY* are also involved in second loop, the morning loop, with the morning-phased genes *PRR7* and *PRR9. PRR7* and *PRR9* are induced by CCA1 and LHY [26] while PRR7 and PRR9 inhibit the expression of *CCA1* and *LHY* [27]. In the third loop, the evening loop, *GIGANTEA* (*GI*), which is negatively regulated by CCA1, LHY and TOC1, was thought to positively regulate *TOC1* in combination with an unknown factor Y [14], [15], [17]. However, Pokhilko *et al.* [20] incorporates the “evening complex” (EC) [28] containing the ELF3 (EARLY FLOWERING 3) [29], ELF4 and LUX proteins into their circadian model and identified a previously unknown connection between GI and *TOC1*. The evening complex binds different target genes, such as *TOC1, GI, PRR9* and *LUX*, and inhibits their expression making the EC an important part of the evening loop. The GI protein is hypothesised to down-regulate the EC complex, causing expression of ECs target genes to increase since inhibition from EC is released, implicating an indirect positive regulation of GI on evening expressed genes such as *TOC1*, something that has also been predicted from simulations [20].

In addition to the main loops described above post-transcriptional factors regulating degradation of specific clock genes are crucial to retain a normal circadian rhythm. A family comprising the F-box proteins ZEITLUPE (ZTL) [30], LOV KELCH PROTEIN2 (LKP2) [31] and FLAVIN BINDING, KELCH REPEAT, F-BOX (FKF1) [32], which all contain three specific domains; the LOV (light, oxygen, or voltage) domain, kelch repeats and an F-box domain, have been shown to be important for normal clock function [30]–[32]. The LOV domain is responsible for blue-light photosensing (reviewed in [33]) and the F-box domain is necessary for ZTL to enable the association with the Skp/Cullin/F-box (SCF) class of E3 ubiquitin ligases, which binds different target genes and degrade them [34]. The kelch repeats have been shown crucial for normal protein binding in both the LOV and F-box domains [35]. Taken together this indicates that the protein family is involved in blue light sensing and protein degradation. In support of this hypothesis, all family members, in particular ZTL, are involved in degradation of TOC1 and PRR5 via ubiquitination [36]–[38]. Further, ZTL interacts with GI in blue light, causing dual stabilization of the proteins. The stabilization results in an accumulation of ZTL in the afternoon [39] despite the lack of rhythmic expression of the gene [30]. After dusk the ZTL-GI interaction weakens and thereby ZTL mediates the degradation and sharpening of the rhythmic expression of TOC1, which is important for a normal circadian rhythm [39].

In summary, the circadian clock in Arabidopsis is a complex system of feedback loops and interactions on different levels, which still is far from completely elucidated, and new knowledge is constantly obtained. The core of the clock does however seem to be confined to a limited set of genes and homologs to several of these have been identified in plant species other than Arabidopsis. For example, homologs to a majority of Arabidopsis core clock genes have been identified in both other eudicot species, such as poplar [40], chestnut [41] and pea [42], [43], and in more distantly related monocot species rice [44], barley [45], *Lemna spp.* [46] and *Brachypodium distachyon* [47]. In some of these studies both functional and expression data support a role of the identified genes in the circadian clock.

Outside angiosperms less data is available and both in gymnosperms and non-vascular plants research efforts have mainly been confined to identification of putative homologs as well as their expression patterns and data on protein and gene function is still limited. The model bryophyte *Physcomitrella patens* seems to only harbour genes corresponding to the morning phase loop [48]. A recent study in the gymnosperm Norway spruce identified and characterized expression pattern for homologs to circadian clock genes. Phylogenetic analyses showed that genes corresponding to all three loops of the Arabidopsis clock model were present in Norway spruce. Furthermore, the spruce homologs displayed diurnal cycling under photoperiodic conditions, but surprisingly the rhythm of mRNA levels for all genes was rapidly dampened under free-running conditions. These data suggests that a network similar to that of the Arabidopsis circadian clock was present before the divergence of angiosperms and gymnosperms. However, the lack of a detectable circadian rhythm for Norway spruce homologs indicate that differences in their protein function and/or their regulation have evolved (Gyllenstrand *et al.* unpublished).

Here we functionally characterize Norway spruce putative homologs to four important Arabidopsis core clock genes namely *PaGI*, *PaZTL*, *PaPRR1* and *PaCCA1*. Homologs to all these genes are central clock components in Arabidopsis and together they represent all identified clock loops as well as the post-transcriptional part of the clock. For three of the genes *PaGI*, *PaZTL* and *PaCCA1* our result show that they can mimic the function of the corresponding gene in Arabidopsis suggesting that their protein function is at least partially conserved between angiosperms and gymnosperms. For, *PaPRR1*, the results are less clear and further research is needed. Nevertheless, our results indicate that the circadian clock in gymnosperms might have a similar basic structure as in angiosperms.

## Materials and Methods

### Phylogenetic analyses

Putative spruce circadian clock homologs were identified through tblastn searches against the spruceEST database at PlantGDB (http://www.plantgdb.org/) using Arabidopsis genes as queries. Full-length genes were obtained by rapid amplification of cDNA ends (Gyllenstrand *et al.* unpublished). Predicted amino acid sequences of putative spruce clock genes and homologous Arabidopsis genes were aligned using Clustal W [49]. Phylogenetic reconstructions were done using PhyML 3.0 [50] and visualized using FigTree 1.2.3 (http://tree.bio.ed.ac.uk/software/figtree/). A Blast search using *PaGI* as query resulted in only one significant hit in the Arabidopsis genome (at1g22770, *GIGANTEA*, e-value 0.0), which was thus considered as the closest homolog.

### Plasmid construction and plant material

Arabidopsis (*Arabidopsis thaliana*) ecotype Columbia (Col) plants expressing the *PaPRR1*, *PaGI*, *PaCCA1* and *PaZTL* genes were produced using Gateway**®** technology (Invitrogen, Carlsbad, CA, USA). The *PaGI* gene was also introduced into the Arabidopsis *gi2* mutant (described in [21]). The complete cDNA was amplified using gene specific primers with *attB*-sites (Table S1). The PCR products were cloned into the pDONR 221 vector (Invitrogen). The destination vector pMDC32 [51] was used to construct 35S*::PaPRR1*, 35S*::PaGI*, 35S*::CCA1* and 35S*::PaZTL* plasmids. The constructs were then transformed into *Agrobacterium tumefaciens* GV3101 pMP90 using the freeze-thaw method. *Agrobacterium tumefaciens*-mediated transformation of Arabidopsis (Col) and *gi2* plants was performed using the floral dip method [52] by The Uppsala Transgenic Arabidopsis Facility and homozygous lines were obtained through selection for three generations. The transgenic Arabidopsis lines obtained were designated *PaPRR1-ox* (for simplicity termed overexpressors), *PaGI-ox*, *gi2-PaGI-ox, PaCCA1-ox* and *PaZTL-ox*. Spruce sequences have been deposited at Genbank under accession no.: KC311521- KC311524.

### Flowering time measurements

Seeds were always sterilized in 10% chlorine and 70% ethanol and stratified in darkness for two to four days at 4°C before use. Arabidopsis (Col) was used as wild type in all experiments. Seeds were planted directly on soil in a randomized manner with 24 pots in each tray. The seedlings were grown under ∼150 µmol m^−2^ s^−1^ cool white fluorescent light in long day (LD), 18 h light/6 h dark in 22°C for *PaZTL-ox* and *PaPRR1-ox* and 16 h light/8 h dark in 22°C for the remaining transformants, or in short day (SD), 9 h light/15 h dark in 22°C during the day and 15°C during the dark period. The flowering time, when the first flower became visible in the rosette, was measured as number of days after sowing and all rosette leaves were counted. Statistical tests comparing each transformed line with control were computed using an ANOVA post-hoc test (Tukey's honestly significant difference test). Calculations were conducted with the aov and TukeyHSD functions in R [53].

### Gene expression analyses

Seeds were sterilized in 10% chlorine and 70% ethanol before stratification in darkness for two to four days in 4°C. Seedlings were grown on Murashige and Skoog (MS) plates with 0.8% plant agar in 22°C under ∼150 µmol m^−2^ s^−1^ in LD (16 h light/8 h dark) to determine the level of expression of the transgene or in 12 h light/12 h dark (12L/12D) cycles. To test for changes in the expression of endogenous genes in the transformants, time series experiments were conducted where seedlings were entrained in 12L/12D followed by transfer to constant light zeitgeber time 0 (ZT0). For *PaZTL-ox* the 12 day old seedlings were sampled every fourth hour (ZT25-49) during one day and immediately snap frozen in liquid nitrogen. *PaCCA1-ox, PaPRR1-ox* and *gi2-PaGI-ox* plants were also entrained in 12L/12D and after 16 days sampled every forth hour for two days, starting directly after transfer to constant light (ZT0-ZT48). The tissue was homogenized with metal balls in a shaker (Retsch MM 300, Haan, Germany). Total RNA was isolated using Qiagen RNeasy® Mini Kit (Qiagen, Hilden, Germany) in accordance with manufacturer's recommendations. The cDNA was synthesized from 0.5 µg total RNA using Superscript III™ Reverse Transcriptase (Invitrogen) following the manufacturer's instructions. Quantitative real-time RT-PCR (qPCR) was performed on either a MyIQ Real-Time PCR Detection System (Bio-rad, Hercules, CA, USA) or an Eco™ Real-Time PCR System (Illumina, San Diego, CA, USA). For MyIQ each reaction was performed in duplicate containing 12 µl DyNAmo™ Flash SYBR®Green (DyNAmo™ Flash SYBR®Green qPCR kit, Thermo Scientific, Waltham, MA, USA), 0.5 mM of each primer and 4.75 µl cDNA (diluted 1∶100) in a total volume of 23.75 µl. For Eco™ each reaction was performed in duplicate containing 5 µl DyNAmo™ Flash SYBR®Green (Thermo Scientific), 0.5 mM of each primer and 4 µl cDNA (diluted 1∶100) in a total volume of 10 µl. Alphabot (Alphahelix Technologie AB, Uppsala, Sweden) performed all pipetting prior to runs in the Eco™. For both systems the thermal conditions used were 95°C for 7 minutes followed by 40 cycles of 95°C for 10 seconds and 60°C for 30 seconds. The primers used are listed in Table S2 and S3. For plotting the time series experiments, expression values were calculated as 2^ΔCT values (CTcontrol – CTtarget)^ using Arabidopsis *α-tubulin* as endogenous control gene. For comparative measurements of expression levels in the transgenic lines, PCR efficiency for each gene was estimated and included in expression calculations, according to the formula 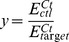, where *E_ctl_* and *E_target_* are estimated PCR efficiencies for the reference gene (*α-tubulin*) and the target gene, respectively, and Ct is the threshold cycle for each gene. PCR efficiencies were calculated from raw fluorescence data using the qPCR package in R [53].

### Leaf movement analysis

Analysis of leaf movement was performed as previously described [54] with slight modifications. Briefly, seeds from transgenic lines and wild type (Col.) were surface sterilized in 10% chlorine and 70% ethanol and stratified for two to four days in 4°C. Individual seedlings were grown on MS medium with 3% sucrose in glass jars in 22°C under ∼25 µmol m^−2^ s^−1^ constant cool white fluorescent light for seven days before entrainment in 12 h light/12 h dark (12L/12D) cycles for five to six days. Pictures were taken every 20 to 60 minutes in constants light for at least seven days and the leaf movements were tracked using ImageJ [55]. Period lengths were determined by fast Fourier transformed non-linear least-square analysis using the Biological Rhythm Analysis Software System (BRASS) 3.0 package (http://millar.bio.ed.ac.uk/), according to the developers instructions.

### Hypocotyl length assays

Sterilized seeds were grown on 0.5× MS plates with 0.8% plant agar and stratified for 3 days. Seedlings were subjected to a light pulse, ∼150 µmol m^−2^ s^−1^ cool white fluorescent light 22°C, for four hours and thereafter kept in darkness for 20 hours. The seedlings were subjected to red light with the indicated fluency rates for 72 h before measurements of the hypocotyls. The *PaZTL-ox* experiment was by necessity performed at higher fluency rates than for other transformants due to the light source available at the time. Controls were constantly kept in darkness after the light pulse. Seedlings were photographed together with a ruler and measured using the software ImageJ [55]. To test for differences in light response between transgenic lines and their respective untransformed lines, a linear model including a transformation of hypocotyl length as the dependent variable, and line, fluency rate and their interaction as independent variables. The interaction terms were used to test for differences in light sensitivity between each transformed line and the control line. Calculations were conducted with the lm function in R [53]. Hypocotyl length (L) was transformed as L′ = 1/(L+0.05).

## Results

### Arabidopsis transformants expressing spruce clock homologs

**Figure 1:**
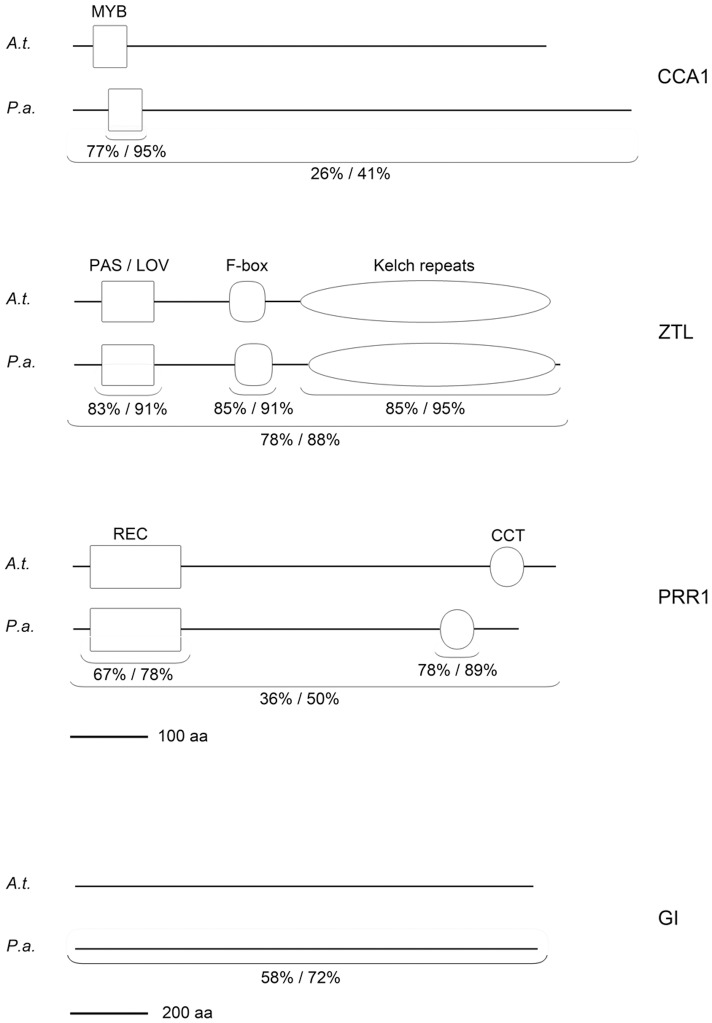
Domain structure of CCA1, ZTL, PRR1 and GI. Protein domain structure in Arabidopsis (*A.t.*) and Norway spruce (*P.a.*). The amino acid similarity in the different domains is stated below each domain and is given as identical amino acids/similar amino acids in percentage. The similarity between the two proteins is given in the same way as for the domains. The domain position and similarity percentage were retrieved using BLAST alignment tool at the NCBI homepage and the whole protein similarity was calculated using Emboss Needle – Pairwise sequence alignment.

Norway spruce homologs of four genes that correspond to important core clock components in Arabidopsis; *PaGI*, *PaCCA1*, *PaZTL* and *PaPRR1* were expressed in Arabidopsis. Blast searches and phylogenetic analyses clearly supported that their closest homologs in Arabidopsis are *GI*, *CCA1*/*LHY*, *ZTL/LKP2* and *PRR1*, respectively (Figure S1; for PaGI only one significant blast hit was obtained). Excluding GI, the corresponding proteins contain defined domains important for their function and these are, in comparison with the Arabidopsis proteins, largely conserved in spruce (Figure 1). *Pa*ZTL is highly conserved throughout the whole protein while *PaCCA1* and *Pa*PRR1 are more divergent overall but have a high degree of conservation in the identified important domains. Although *Pa*GI lacks defined domains the overall amino acid similarity is relatively high.

**Figure 2:**
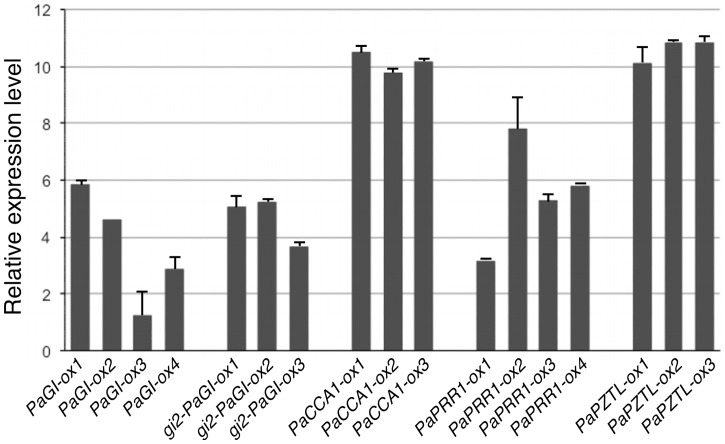
Quantification of expression levels of transgenes by qPCR. Five different Arabidopsis transformants expressing four different spruce genes were created using *Agrobacterium*-mediated DNA transfer. Estimated expression levels were log2 transformed. See Materials & Methods for details about calculation of expression levels. Arabidopsis *α-tubulin* was used as endogenous control. Error bars indicate SE values.

The coding region of *PaGI*, *PaCCA1*, *PaZTL* and *PaPRR1*, were fused to the Cauliflower Mosaic virus (CaMV) 35S promoter and transformed into wild type Arabidopsis (Columbia) plants using *Agrobacterium*-mediated DNA delivery. *35S::PaGI* was also transformed into the *gi2* mutant, which has a severely truncated GI protein and altered flowering time in both long (LD) and short days (SD) [21]. Homozygous transgenic lines were obtained for all transformations and for each construct three to four lines were selected for further characterization based on flowering time phenotype and/or relative expression level. Seedlings were grown in LD conditions to determine the expression level for all lines with real-time RT-PCR (qPCR). All selected lines showed expression of the transgene (Figure 2), and for simplicity they were named *gi2-PaGI-ox, PaGI-ox, PaCCA1-ox, PaZTL-ox, PaPRR1-ox*. The *PaCCA1* and *PaZTL* expressing plants (*PaCCA1-ox1-3* and *PaZTL-ox1-3*, respectively) displayed high and similar expression levels of the transgene compared to the other transformants.

### Several putative spruce clock genes affect flowering time in Arabidopsis

**Figure 3:**
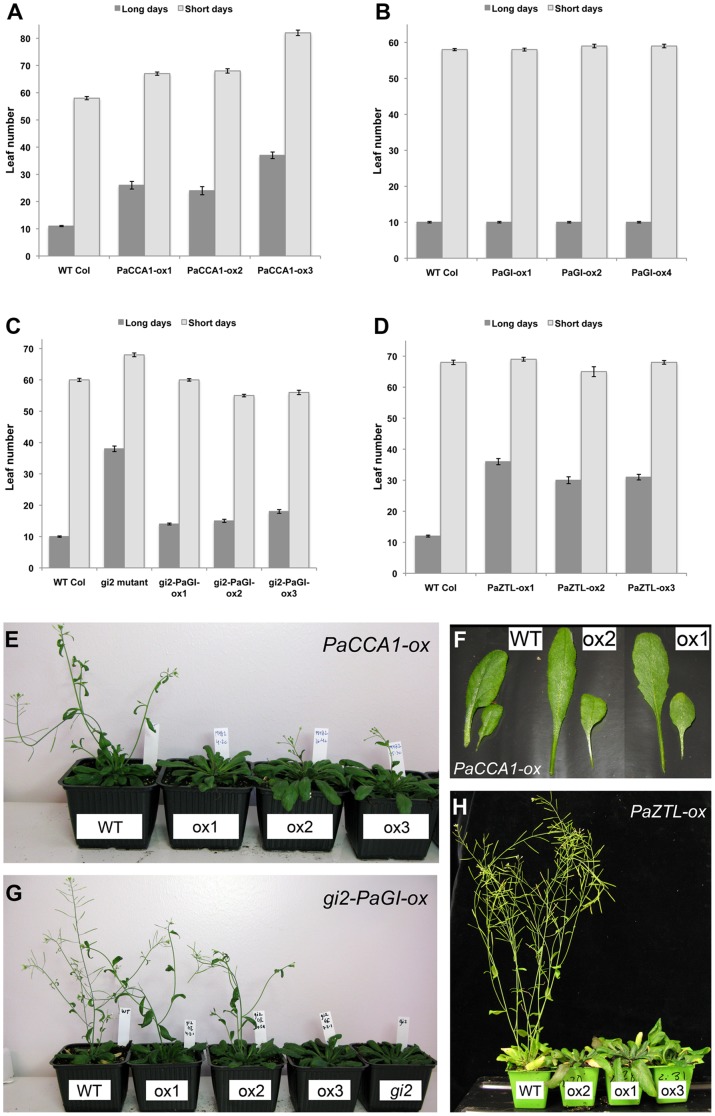
Flowering time was altered for some of the Arabidopsis transgenic lines. Selected lines expressing (A) *PaCCA1*, (B) *PaGI*, (C) *PaGI* in the *gi2* mutant and (D) *PaZTL* were grown in LD (18 h light/6 h dark or 16 h light/8 h dark) and SD (9 h light/15 h dark) and flowering time was assessed as number of rosette leaves when the first flower bud was visible in the rosette. Pictures of representative plants grown in LD for 46 days were taken for (E) *PaCCA1*, (G) *PaGI* in the *gi2* mutant and (H) *PaZTL*. In *PaCCA1-ox* the petioles were longer than the wt (F). Error bars indicate SE values.

Arabidopsis is a facultative long day plant meaning that transition from vegetative state to reproduction (flowering) occurs much earlier in long days compared to short days. Flowering time is coupled to the circadian clock through the control of *CONSTANS* (*CO*), which in turn promotes the floral regulator *FLOWERING LOCUS T* (*FT*) [56]. We examined flowering time in LD (18 h light/6 h dark or 16 h light/8 h dark) and SD (9 h light/15 h dark) for all the created transgenic lines as well as wild type plants (Figure 3). In all experiments, both the number of days to flowering (measured as the day when a flower bud became visible in the rosette) and the number of rosette leaves at flowering were counted. Since these results were consistent only leaf number data is presented here. All *PaCCA1-ox* lines showed significantly delayed flowering time in LD as well as SD (Figure 3A and E; all *P*-values<10^−7^). When *AtCCA1* is overexpressed in Arabidopsis flowering is delayed in both LD and SD [3], [10], consistent with the results from our transformants. The petioles (Figure 3F) and hypocotyls were also clearly longer in the transgenic plants as previously observed for *AtCCA1-ox* [57].

The *PaGI-ox* lines displayed no significant difference in flowering time compared to wild type (Figure 3B; all *P*-values>0.25), but expressing the spruce gene in the Arabidopsis *gi2* mutant (*gi2-PaGI-ox1-3*) resulted in much earlier flowering compared to the *gi2* mutant (Figure 3C and G; all *P*-values<10^−7^), and almost restored flowering time of the mutant line to wild type (wt). However, overexpressors of *AtGI* in wt flower very early in SD [58] in contrast to our *PaGI-ox* lines.

*AtZTL-ox* flower late in LD, up to the point where some overexpressor lines flower simultaneously in LD and SD, but in SD there are no major effects [59]. In *PaZTL-ox* similar patterns, with late flowering, were observed in LD only (Figure 3D and H; all *P*-values<10^−7^), but the effect on flowering time is not as strong as in some *AtZTL-ox* lines.

*PaPRR1-ox* showed no detectable effect on flowering time in either growth condition and the elongated petioles observed in *AtTOC1-ox* [60] could not be observed for *PaPRR1-ox* (data not shown). However, Arabidopsis plants overexpressing *AtTOC1* or *OsPRR1* (the rice (*Oryza sativa) TOC1* homolog) had a retarded growth, and comparable flowering time data has not been reported [44], [60].

### Free-running circadian period is altered in Arabidopsis plants expressing *PaCCA1* and *PaZTL*

One of the outputs of the circadian clock is rhythmic leaf movement and properties of the circadian clock can be estimated by tracking leaf position [54]. To estimate the period of the circadian rhythm in transgenic lines, plants were entrained in 12 h light/12 h dark and then transferred to constant light (LL). Leaf movements were tracked for each plant and the results were used to estimate period length for each transgenic line (Table 1). *PaCCA1-ox* lines were all arrhythmic, while *PaZTL-ox* displayed a shorter period than wt (approximately 21 to 23 hours for the transgenic lines compared to 25 hours for the wt). When overexpressed in Arabidopsis *AtCCA1-ox* become arrhythmic [10] and *AtZTL-ox* seedlings are arrhythmic or have a short period [59], [61]. The period lengths for *PaGI-ox* and *PaPRR1-ox* were not significantly different from wt, in contrast to overexpressors of *AtGI* and *AtPRR1*, which have a shorter period than wt or are arrhythmic [58], [62]. The period of leaf movement of *gi2* is reported to be similar to wt, but due to low amplitude, period is difficult to estimate in the *gi2* mutant [63]. Our estimate of period of *gi2* plants was slightly lower that wt, but accompanied with a large standard error. However, *gi-PaGI-ox* lines displayed a period significantly shorter than wt and *gi2*, indicating that expression of *PaGI* might have a similar effect on circadian rhythm as *AtGI*. The lack of effect on circadian period when expressing *PaGI* in wt might result from insufficient expression levels in these transgenic lines or differences in protein function.

**Table 1:** Circadian period of leaf movement in Arabidopsis lines expressing spruce clock genes.

Construct	Line	Period	SEM	RAE	n
Experiment 1					
WT	Col	25.14	1.10	0.09	8
*PaZTL-ox*	ox1	22.72	0.81	0.12	7
	ox2	21.10	0.02	0.09	4
	ox3	21.63	0.13	0.08	10
Experiment 2					
WT	Col	24.85	0.22	0.17	10
*PaPRR1-ox*	ox1	24.21	0.22	0.16	18
	ox2	24.5	0.18	0.19	20
*PaCCA1-ox*	ox1	Arrhythmic*			20
	ox3	Arrhythmic*			20
*PaGI-ox*	ox2	24.59	0.11	0.23	20
	ox3	24.47	0.19	0.18	19
*gi* mutant	*gi2*	23.6	0.57	0.37	6
*gi2-PaGI-ox*	ox1	22.23	0.36	0.27	16
	ox2	22.05	0.32	0.24	14

Weighted mean circadian periods were estimated as described in Materials and Methods. SEM, standard error of the mean; RAE, relative amplitude error; n, number of rhythmic plants.

* No rhythmic plants were observed for *PaCCA1-ox* lines.

### Red light sensitivity is altered when certain spruce clock genes are expressed in Arabidopsis

**Figure 4:**
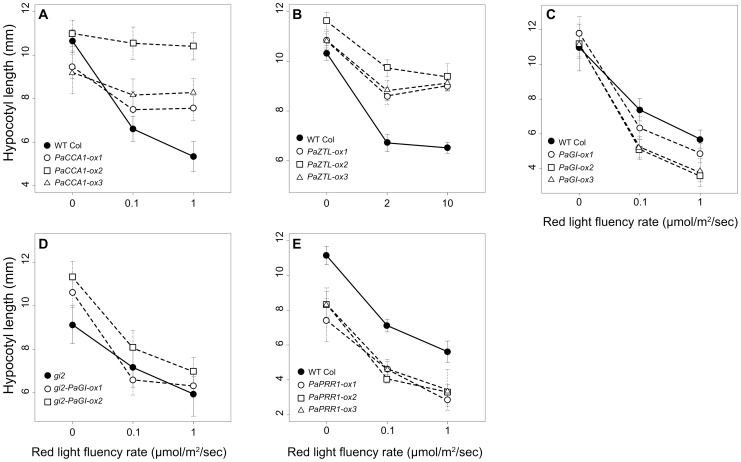
Altered hypocotyl length in red light. Hypocotyl length was affected by red light in some of the Arabidopsis transgenic lines expressing spruce genes. Hypocotyl length of (A) *PaCCA1-ox*, (B) *PaZTL-ox*, (C) *PaGI-ox*, (D) *gi2-PaGI-ox* and (E) *PaPRR1-ox* under red light. Seeds grown on MS-agar plates were stratified and given a four-hour light pulse before transferred to darkness for 20 hours. The seedlings were thereafter subjected to the indicated fluency rate of red light for 72 hours before measurement of the hypocotyls. Controls were, after the light pulse, kept in the dark through the entire experiments. Error bars indicate SE values. Statistical tests are included in Table 2.

Hypocotyl elongation is controlled by an interaction between the circadian system and light signaling [64]. Altered expression of different clock genes can affect hypocotyl growth under different light conditions. Arabidopsis seedlings expressing *PaCCA1* are hyposensitive to red light (all *PaCCA1-ox* have longer hypocotyls than the wt in 0.1 and 1 µmol m^−2^ s^−1^; Figure 4A, Table 2), which is consistent with results from *AtCCA1-ox* and *OsCCA1-ox* both being hyposensitive [44], [65]. When *PaZTL* is expressed in Arabidopsis, the seedlings also become hyposensitive to red light resulting in elongated hypocotyls (Figure 4B, Table 2), again consistent with results from expressing the Arabidopsis homolog (*AtZTL-ox*) and the Rice homolog (*OsZTL1-ox*) [44], [59], [61]. *PaGI-ox* lines showed weak but significant hypersensitive response to red light in wt background (Figure 4C, Table 2), and *PaGI-ox* lines in the *gi2* background showed the same tendency, but were not significantly different from *gi2* (Figure 4C and D, Table 2). The non-significant results from the *gi2-PaGI-ox* could partly be due to problems with germination in those lines under the applied conditions. Still, the apparent hypersensitive response in the *PaGI-ox* lines is in agreement with that of *AtGI-ox* plants [58]. No significant effect on red light response was observed in the *PaPRR1-ox* lines (Figure 4E, Table 2), in contrast to the effect observed in *AtTOC1/PRR1-ox* plants that are hypersensitive to red light [44], [66].

**Table 2:** Statistical tests for altered sensitivity to red light among Arabidopsis lines expressing spruce clock genes.

Line	*P*-slope*	Model *R^2^* **	Sensitivity***
*PaCCA1-ox1*	5×10^−7^	0.59	Hypo
*PaCCA1-ox2*	4×10^−14^		Hypo
*PaCCA1-ox3*	1×10^−9^		Hypo
*PaZTL-ox1*	1×10^−9^	0.60	Hypo
*PaZTL-ox2*	5×10^−9^		Hypo
*PaZTL-ox3*	1×10^−10^		Hypo
*PaGI-ox1*	1×10^−2^	0.79	Hyper
*PaGI-ox2*	2×10^−4^		Hyper
*PaGI-ox3*	7×10^−4^		Hyper
*gi2-PaGI-ox1*	4.8×10^−2^	0.59	Hyper
*gi2-PaGI-ox2*	6.7×10^−2^		(Hyper)
*PaPRR1-ox1*	7×10^−2^	0.70	
*PaPRR1-ox2*	9×10^−1^		
*PaPRR1-ox3*	5×10^−1^		

Transgenic lines were compared against the corresponding Arabidopsis lines without transgene.

* *P*-value calculated from the interaction term Fluency rate x Line in a linear model including Line and Fluency rate, see materials and methods for details.

** Adjusted R^2^ (coefficient of determination) indicating model fit.

*** Hypo and Hyper indicate that ectopic expressor lines were hypo- or hypersensitive to red light as compared to untransformed lines. Parentheses mark non-significant effects.

### Arabidopsis clock genes are affected by expression of spruce clock genes

Since the circadian system consists of several interconnected loops the participating genes generally affect each other's expression. Expression of the core Norway spruce clock genes described here in Arabidopsis is therefore expected to alter the expression of other Arabidopsis clock components if their function is conserved. Selected transgenic lines were grown on agar plates and entrained in 12 h light/12 h dark for 12 (*PaZTL-ox*) or 16 days (remaining transformants) and thereafter transferred to constant light (LL).

**Figure 5:**
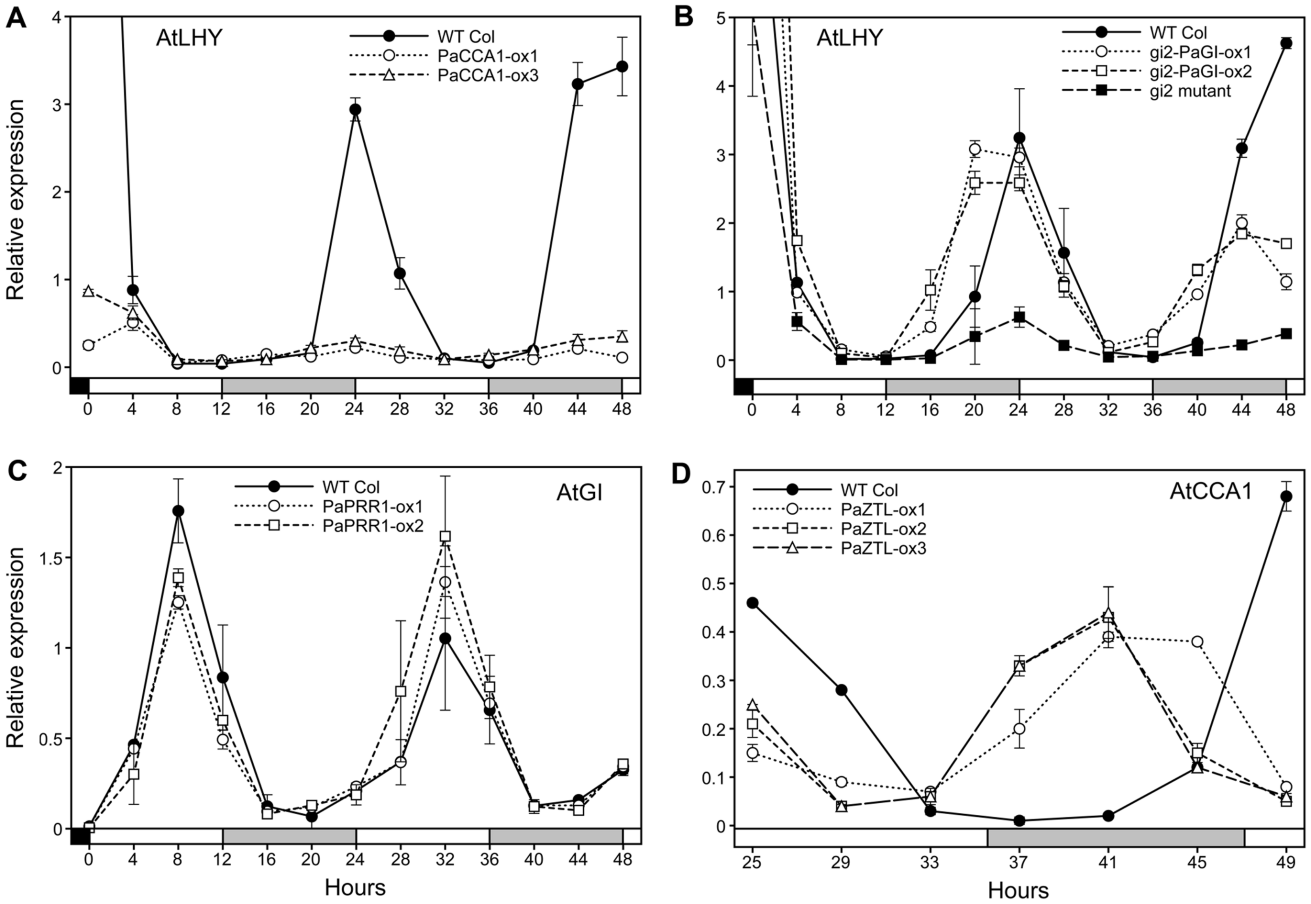
The expression of endogenous clock genes was altered in some transformants. Selected transgenic lines were entrained in 12 h light/12 h dark for 12 (*PaZTL-ox*) or 16 days (the remaining transformants) before subjected to constant light (LL). *PaCCA1-ox*, *gi2*-*PaGI-ox* and *PaPRR1-ox* were sampled directly after transition to LL (ZT0-ZT48), while *PaZTL-ox* was sampled after 25 hour in LL (ZT25-ZT49). The expression of *AtLHY, AtGI* and *AtCCA1* were measured by qPCR in the (A) *PaCCA1-ox*, (B) *gi2-PaGI-ox*, (C) *PaPRR1-ox* (D) *PaZTL-ox* lines, respectively. Relative expression values were calculated as 2^ΔCT values (CTcontrol – CTtarget)^ using Arabidopsis *α-tubulin* as endogenous control. Error bars indicate SE values.

*PaCCA1-ox*, *gi2-PaGI-ox* and *PaPRR1-ox* lines were sampled directly after transition to LL (sampling time expressed as zeitgeber time, ZT0-ZT48), while *PaZTL-ox* lines were sampled after 25 hour in LL (ZT25-ZT49). In the *PaCCA1-ox* lines *AtLHY* expression was strongly repressed and the circadian rhythm was abolished (Figure 5A), and *AtTOC1* (Figure S2A) show a strongly dampened rhythm. In *AtCCA1-ox* plants entrained under the same conditions followed by transfer to LL the *AtLHY* gene was expressed at low levels and transcript abundances did not cycle. Likewise, the rhythm of the *AtTOC1* gene was impaired and showed dampened cycling [10], [44].

*gi2* mutants display a largely constant and low expression of *AtLHY* and *AtFT* (Figure 5B and Figure S2B, respectively) while the wt exhibit a rhythmic expression with a peak before dawn for *AtLHY* (Figure 5B) and after dusk for *AtFT* (Figure S2B). A rhythmic expression is restored in *gi2-PaGI-ox*, suggesting that *PaGI* can partly rescue clock related phenotypes of *gi2*. Overexpressors of *AtGI* in Arabidopsis also showed a rhythmic and increased expression of *AtFT*, at least in SD (10 h light/14 h dark) [58].

For the *PaPRR1-ox* lines, the expression of *AtGI* (Figure 5C), *AtPRR9* and *AtLHY* (Figure S2C and D) were examined but none of these genes were affected by the *PaPRR1* overexpression. In contrast, overexpression of *AtTOC1/PRR1* in Arabidopsis has a large impact on the expression of other clock genes, including *AtGI*, *AtPRR9*, and *AtLHY* [44], [60].

The phase of expression was dramatically shifted for *AtCCA1* in the *PaZTL-ox* lines (Figure 5D) resulting in peak expression at the middle of the dark period in contrast to wt where the expression peaks at subjective dawn. Under the same experimental conditions such a phase shift [59] or a strong dampening of the rhythmic expression of *AtCCA1* [44] has been observed in *AtZTL-ox* lines.

## Discussion

Limited information is currently available about circadian clock mechanisms in plant species outside angiosperms [67]. Based on sequence similarity and patterns of gene expression current data suggests that plant evolution has been characterized by an increased complexity of circadian clock mechanisms [48], [68]. In the unicellular green alga, *Ostreococcus tauri*, a single loop consisting of *CCA1* and *TOC1* homologs has been reported [69]. In the moss, *Physcomitrella patens*, orthologs to the core clock genes *TOC1, GI* and *ZTL* are lacking and therefore two of the loops in the Arabidopsis circadian system appear to be missing [48]. However, one of the loops has been proposed to remain, containing *PpCCA1a* and/or *PpCCA1b* and the *PpPRR1-4* [48]. This would suggest that *CCA1* homologs were present before the split between the bryophytes (e.g. moss) and vascular plants. One of the *PpPRR*s, *PpPRR2*, has been shown to affect circadian related phenotypes, such as flowering time, period and hypocotyl length, when expressed in Arabidopsis [70] although it is unclear if it is an ortholog of *TOC1* [48]. Evidence for the existence of orthologs to *TOC1* as well as the other two main evening loop genes *GI* and *ZTL* also in *Selaginella moellendorffi* [48] suggests that a three-loop mechanism similar to the one in angiosperms could have evolved early in the evolution of vascular plants. Such a three-loop mechanism is generally supported in angiosperms, both in monocots and dicots (see for instance [40], [41], [42], [43], [44], [46], [47], [71]). In Norway spruce, homologs to genes involved in the evening loop (*GI*, *TOC1*, *ZTL*) were identified, as well as homologs to genes of the morning loop (*TOC1* and *CCA1/LHY*) suggesting that gymnosperms might have a three-loop mechanism similar to the clock in angiosperms. Expression data further corroborated that these genes are part of a clock-like network in Norway spruce. The spruce homologs described in this paper show diurnal cycling of transcript abundances, except *PaZTL*. In Arabidopsis *ZTL* is also constitutively expressed and not regulated by light [30]. However, cycling of the spruce genes was rapidly dampened in free-running conditions in contrast to observations of clock gene expression in most other plant species (Gyllenstrand *et al.* unpublished). Thus, to unravel the diversity and evolution of plant circadian clock functional studies of clock genes outside model angiosperms are needed.

The evaluation of circadian clock related phenotypes in conifers is hampered by a lack of established assays of clock outputs. The production of transgenic conifers is also a very slow process. For these reasons, expression of conifer clock genes in Arabidopsis is a logical first step to investigate the conifer circadian clock, and to evaluate the degree of evolutionary conservation of circadian clock mechanisms. We took advantage of the detailed knowledge of clock function and gene interaction in Arabidopsis and created transgenic lines ectopically expressing the putative clock genes from Norway spruce. Even though phenotypic alterations in ectopic expression lines must be interpreted with caution, our data suggests that at least some components of the Norway spruce circadian clock share similar functions as their homologs in angiosperms.

Arabidopsis homologs of *PaCCA1*, *PaGI, PaZTL* and *PaPRR1* all have central functions in the Arabidopsis circadian system and the sequences of the corresponding predicted spruce proteins are highly conserved (Figure 1). Expression of three of the spruce putative clock genes in Arabidopsis affected some phenotypes relating to clock gene function, with *PaCCA1* and *PaZTL* showing the strongest effects. All the observed phenotypes were as expected from those seen when overexpressing the corresponding Arabidopsis genes [3], [10], [44], [57], [59], [61], [65]. For *PaCCA1-ox* lines these included late flowering, arrhythmicity, altered expression of other clock genes and hyposensitivity to red light. Similar results were obtained for lines expressing *PaZTL*, except that the plants still possessed a circadian rhythm albeit with a significantly shorter period. Results largely consistent with those when overexpressing the endogenous gene in Arabidopsis were also obtained for *PaGI-ox* in the mutant line *gi2*, including a restoration of flowering time, increased sensitivity to red light, a shortened circadian rhythm and increased abundance of *LHY* and *FT* mRNA [58], [63]. In contrast, lines expressing *PaGI* in wt showed no effect on circadian rhythm or flowering time, and only a suggestive effect of increased sensitivity to red light. No effects on the studied phenotypes were observed in *PaPRR1-ox* lines.

There may be several reasons for the phenotypic differences observed between spruce genes. The differences could to some extent be due to the expression levels of the transgenes. *PaCCA1-ox* and *PaZTL-ox* lines all showed significantly higher expression levels when compared to the other transgenic lines (Figure 2). The hypothesis that the transgene expression in *PaGI-ox* and *PaPRR1-ox* might not have been high enough to overrule the effects of the endogenous genes is supported by the fact that significant effects were observed in the *gi2-PaGI-ox* lines with a similar level of transgene expression as in *PaGI-ox* lines. Other possible causes include differences in post-transcriptional regulation, and we cannot role out functional divergence of the Norway spruce proteins when compared to their Arabidopsis counterparts as a reason for the variable responses observed for the Norway spruce clock genes.

In conclusion, our results suggest conservation of protein function in at least three central clock components, *PaCCA1, PaGI* and *PaZTL*, indicating that multiple parts of the circadian clock mechanism in gymnosperms are partly conserved when compared to angiosperms. A conserved function of *CCA1* homologs is perhaps not surprising since these genes have a function in circadian clocks even in bryophytes. Our results do not contradict the idea that the circadian three-loop system evolved before the split between angiosperms and gymnosperms. Circadian clock genes seem to play a major role in local adaptation not only in angiosperms but also in gymnosperm species [7]. It is thus of great interest to understand how variation in photoperiod is measured and how these signals are relayed to control traits such as annual growth rhythm in perennial trees. Even though further studies are clearly needed to clarify the regulation and interaction of the circadian clock genes in Norway spruce and other gymnosperms, this study is a key step towards the understanding of the plant circadian system and its evolution.

## Supporting Information

Figure S1 **Phylogenetic reconstructions of spruce circadian clock genes** ***PaCCA1*** **,** ***PaPRR1*** **and** ***PaZTL*** **Arabidopsis homologs.**
(PDF)

Figure S2 **The expression of endogenous genes was examined by qPCR.** Selected transgenic lines were entrained in 12 h light/12 h dark for or 16 days before subjected to constant light (LL). *PaCCA1-ox*, *gi2*-*PaGI-ox* and *PaPRR1-ox* where sampled directly after transition to LL (ZT0-ZT48). The expression of *AtTOC1, AtFT, AtPRR9* and *AtLHY* where measured by qPCR in the (A) *PaCCA1-ox*, (B) *gi2-PaGI-ox* and (C and D) *PaPRR1-ox*, respectively. Relative expression values were calculated as 2^ΔCT values (CTcontrol – CTtarget)^ using Arabidopsis *α-tubulin* as endogenous control. Error bars indicate SE values.
(TIF)

Table S1 Primers used for Gateway**®**-constructs.
(DOCX)

Table S2 Primers used for qPCR experiments.
(DOCX)

Table S3 Primers used to estimate expression in transgenic lines.
(DOCX)
