# Supplementary figures and images for: Conserved Function of Core Clock Proteins in the Gymnosperm Norway Spruce (Picea abies L. Karst)
Source: PLoS One. 2013 Mar 28;8(3):e60110. doi: 10.1371/journal.pone.0060110 (PMC3610754; doi:10.1371/journal.pone.0060110)

Supplemental figure S1

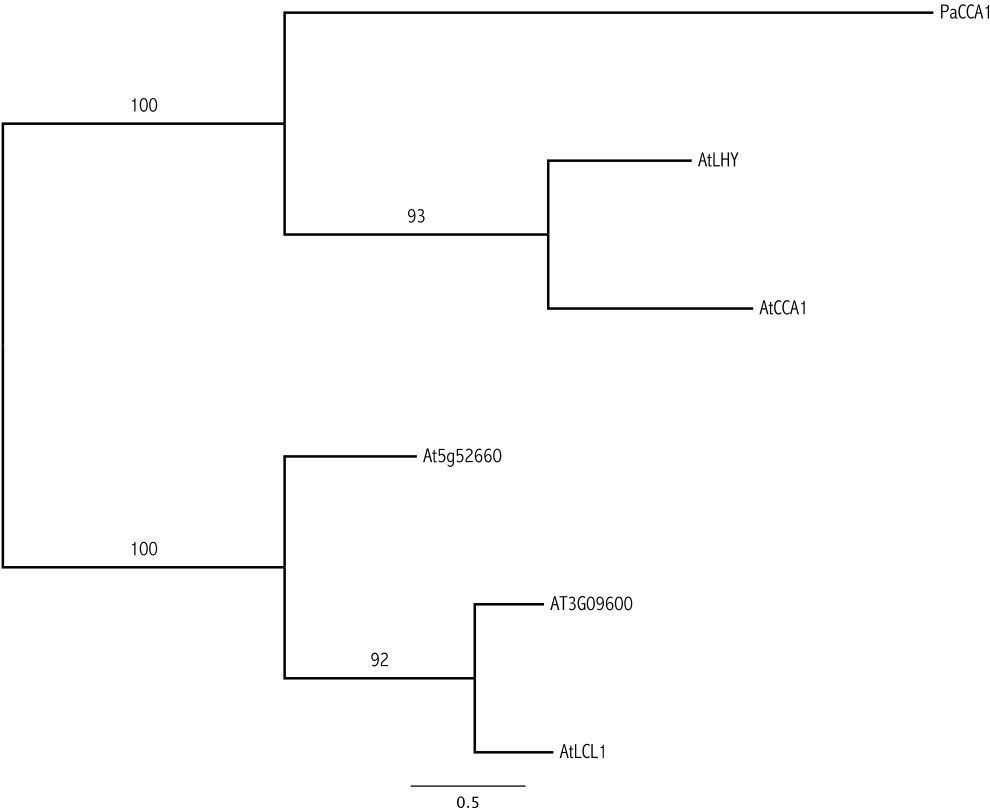

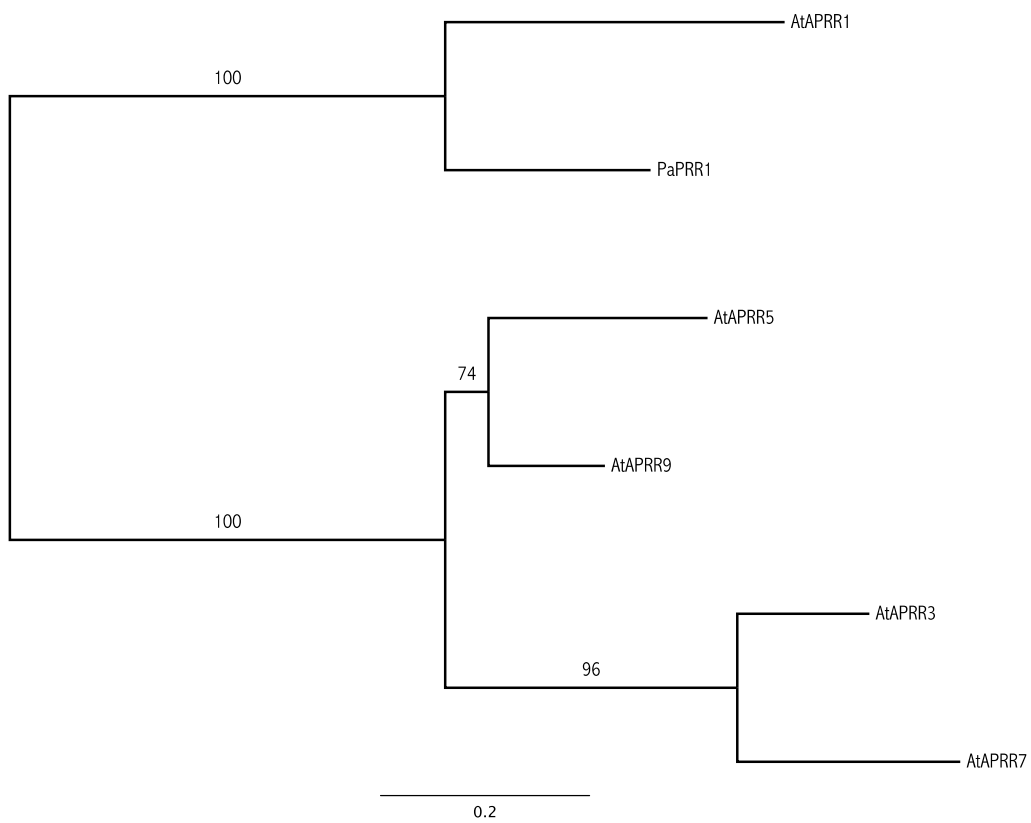

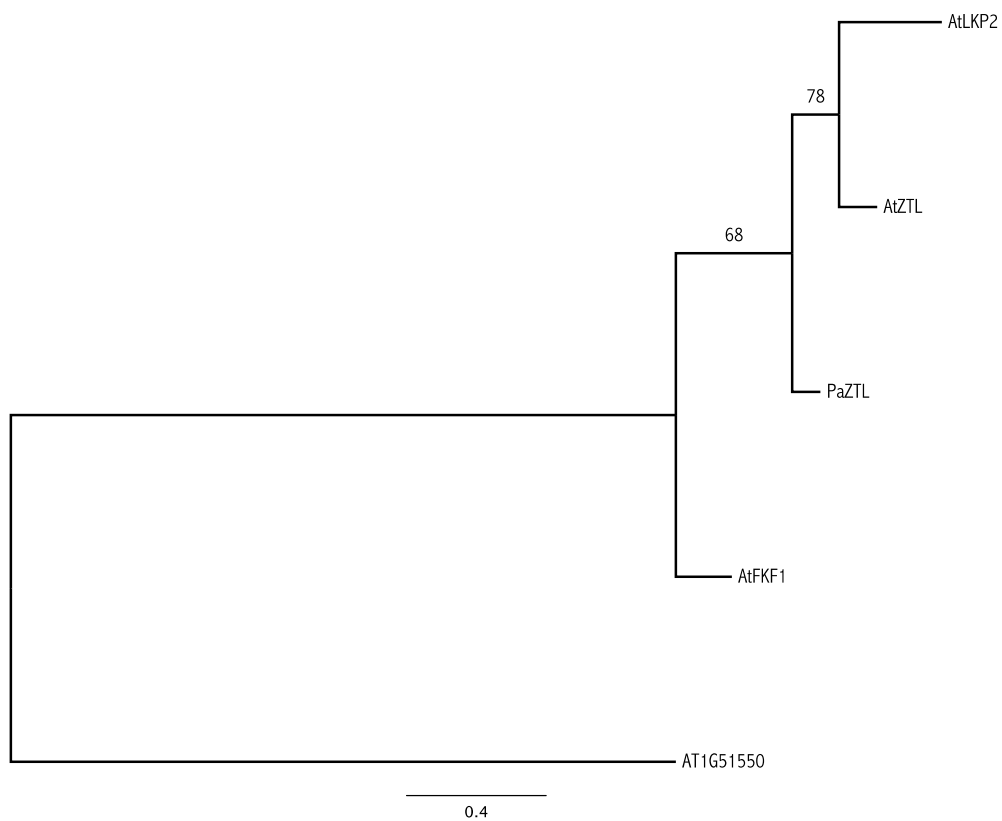

Supplement: Figure S1 — Phylogenetic reconstructions of spruce circadian clock genes PaCCA1 , PaPRR1 and PaZTL Arabidopsis homologs. (PDF) [file pone.0060110.s001.pdf]

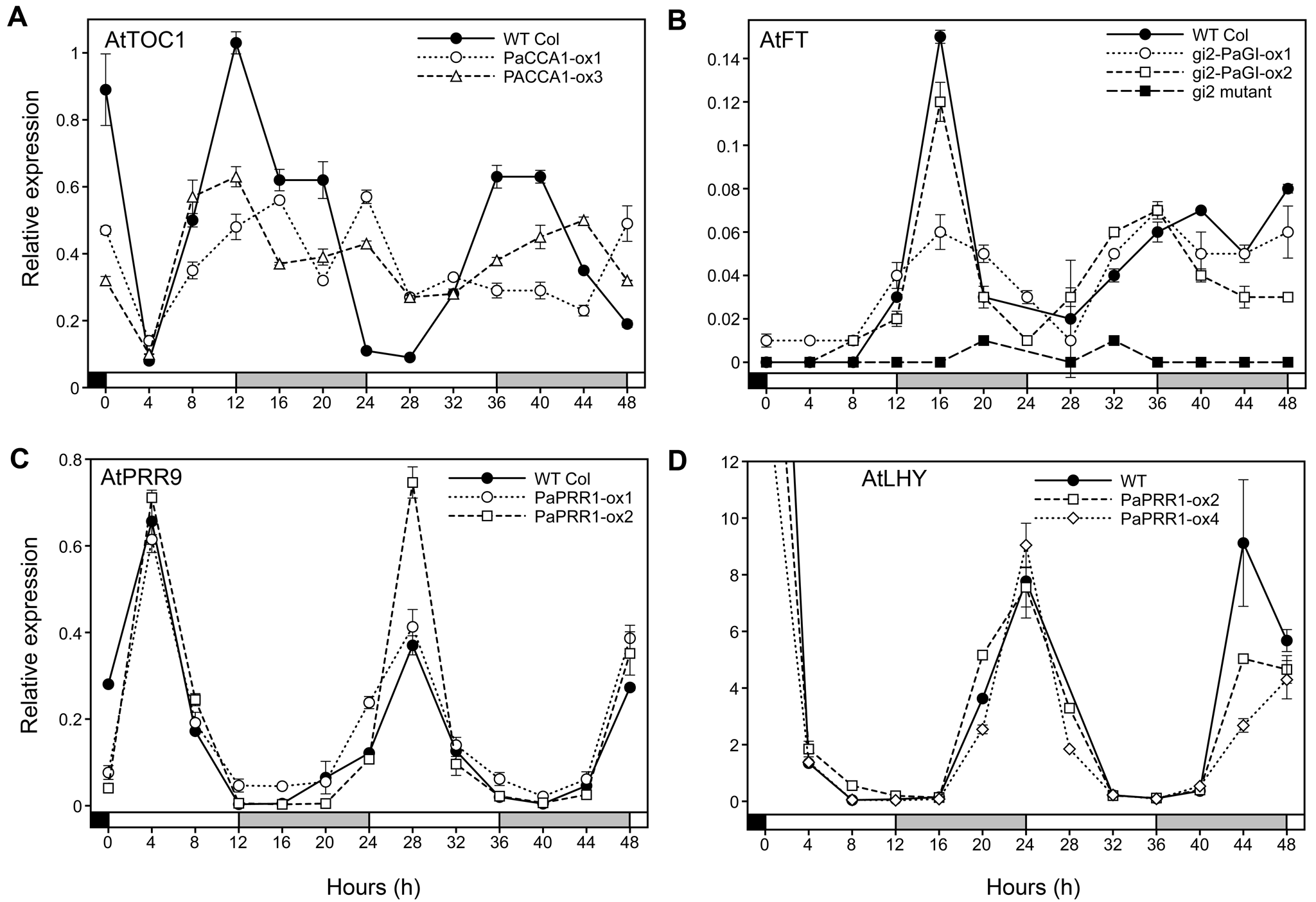

Supplement: Figure S2 — The expression of endogenous genes was examined by qPCR. Selected transgenic lines were entrained in 12 h light/12 h dark for or 16 days before subjected to constant light (LL). PaCCA1-ox, gi2-PaGI-ox and PaPRR1-ox where sampled directly after transition to LL (ZT0-ZT48). The expression of AtTOC1, AtFT, AtPRR9 and AtLHY where measured by qPCR in the (A) PaCCA1-ox, (B) gi2-PaGI-ox and (C and D) PaPRR1-ox, respectively. Relative expression values were calculated as 2ΔCT values (CTcontrol – CTtarget) using Arabidopsis α-tubulin as endogenous control. Error bars indicate SE values. (TIF) [file pone.0060110.s002.tif]
