# Supplementary material for: Conserved Function of Core Clock Proteins in the Gymnosperm Norway Spruce (Picea abies L. Karst)
Source: PLoS One. 2013 Mar 28;8(3):e60110. doi: 10.1371/journal.pone.0060110 (PMC3610754; doi:10.1371/journal.pone.0060110)
Supplement: Table S1 — Primers used for Gateway®-constructs. (DOCX) [file pone.0060110.s003.docx]

**Table S1.** Primers used for Gateway-constructs.

| Gene | Primers 5´→ 3´ |
| --- | --- |
| *PaGI* | attB1 -GGGGACAAGTTTGTACAAAAAAGCAGGCTTCATGTCTATATCAGAGCAAAAGTG  attB2 - GGGGACCACTTTGTACAAGAAAGCTGGGTGTCAAACATTTGCAGAGAAAC |
| *PaCCA1* | attB1 - GGGGACAAGTTTGTACAAAAAAGCAGGCTTCATGAAGATGTCTCTGCCT  attB2 - GGGGACCACTTTGTACAAGAAAGCTGGGTTGAGCGATGTTCAATGTAAC |
| *PaPRR1* | attB1 - GGGGACAAGTTTGTACAAAAAAGCAGGCTGCATGGGGAAGGGTAGTATATC  attB2 - GGGGACCACTTTGTACAAGAAAGCTGGGTTCTATCTTTGATATTGAATAGC |
| *PaZTL* | attB1 - GGGGACAAGTTTGTACAAAAAAGCAGGCTTCATGGAGTGGGACAGTGGTTCC  attB2 - GGGGACCACTTTGTACAAGAAAGCTGGGTGCTAATCTAACCCATGTTTGC |
