# Supplementary material for: Conserved Function of Core Clock Proteins in the Gymnosperm Norway Spruce (Picea abies L. Karst)
Source: PLoS One. 2013 Mar 28;8(3):e60110. doi: 10.1371/journal.pone.0060110 (PMC3610754; doi:10.1371/journal.pone.0060110)
Supplement: Table S2 — Primers used for qPCR experiments. (DOCX) [file pone.0060110.s004.docx]

| Gene | Primers 5´→ 3´ |
| --- | --- |
| *AtTUB* | FWD - ACCACTCCTAGCTTTGGTGATCTG  REV - AGGTTCACTGCGAGCTTCCTCA |
| *AtCCA1* | FWD - TCTGTGTCTGACGAGGGTCGAATT  REV - ACTTTGCGGCAATACCTCTCTGG |
| *AtLHY* | FWD - ACGAAACAGGTAAGTGGCGACATT  REV - TGGGAACATCTTGAACCGCGTT |
| *AtTOC1* | FWD - TCTTCGCAGAATCCCTGTGAT  REV - GCTGCACCTAGCTTCAAGCA |
| *AtFT* | FWD - TACGAAAATCCAAGTCCCACTG  REV - AAACTCGCGAGTGTTGAAGTTC |
| *AtGI* | FWD - CTGTCTTTCTCCGTTGTTTC  REV - ATCAACAACCTGTCTCCATC |
| *AtPRR9* | FWD - CTGATGCGTCGGCCTTCTC  REV - CGACGGCTTTTTCTGCTGACT |

**Table S2.** Primers used for qPCR experiments.
