# Supplementary material for: Conserved Function of Core Clock Proteins in the Gymnosperm Norway Spruce (Picea abies L. Karst)
Source: PLoS One. 2013 Mar 28;8(3):e60110. doi: 10.1371/journal.pone.0060110 (PMC3610754; doi:10.1371/journal.pone.0060110)
Supplement: Table S3 — Primers used to estimate expression in transgenic lines. (DOCX) [file pone.0060110.s005.docx]

**Table S3**. Primers used to estimate expression in transgenic lines.

| **Gene** | **Primers 5´→ 3´** |
| --- | --- |
| *PaGI* | FWD - GGCAGAAGGGCTATGGAA  REV - ATGTACTAAGTGCGCGGACA |
| *PaCCA1* | FWD - TTCTCACTCTCAGCGGGGATTC  REV - TGTGTATGGTTTCCTCACCTTTGC |
| *PaPRR1* | FWD - TCATCCTGAGTGCGAGTCAC  REV - TCACTCTTCTTTGGGCACGAG |
| *PaZTL* | FWD - GCCGATTATTTATGTAAACGCAGTG  REV - GGATGCCTTCTCTGTGCAAATG |
| *α*-*tubulin* | FWD - ACCACTCCTAGCTTTGGTGATCTG  REV - AGGTTCACTGCGAGCTTCCTCA |
